# AI-integrated digital breeding for crop improvement

**DOI:** 10.3389/fpls.2026.1929693

**Published:** 2026-08-20

**Authors:** Seungmin Son, Sang Ryeol Park

**Affiliations:** National Institute of Agricultural Sciences, Rural Development Administration, Jeonju, Republic of Korea

**Keywords:** artificial intelligence, big data, digital breeding, *in silico*, multi-omics data, phenomic data

## Abstract

Crop breeding increasingly depends on the effective integration and interpretation of large, heterogeneous datasets spanning genomic, phenotypic, multi-omics, and environmental layers. Conventional breeding approaches are often insufficient to capture the complex relationships among these data or to support timely selection decisions. Digital breeding can help address this limitation by complementing field experimentation, mixed models, and genomic prediction with the integration of biological data and computational prediction throughout the breeding process. In particular, the rapid advancement of artificial intelligence (AI) has improved the analysis of high-dimensional datasets and broadened its application to trait prediction, selection, and breeding design. Here, we review recent developments in AI-enabled digital breeding, encompassing genomic, phenomic, and multi-omics data generation and analysis, predictive modeling, explainable and generative AI, and data-driven breeding decision support. We further discuss emerging AI applications, their current contributions to crop research and breeding, and the major considerations affecting their reliable and practical implementation. Collectively, this review provides a structured understanding of the roles of AI across the digital breeding process and offers guidance for future methodological development and practical application in crop improvement.

## 1 Introduction

Climate change is placing crop production under growing pressure worldwide. Rising temperatures, irregular rainfall, and increasingly frequent extreme weather events expose plants to a broad range of abiotic stresses, including heat, drought, flooding, and salinity (Zandalinas et al., 2021; Pandey and Senthil-Kumar, 2024). These conditions disrupt plant growth, photosynthesis, nutrient acquisition, and reproductive development, often resulting in substantial yield losses. At the same time, climate change can intensify biotic stress by altering the distribution and activity of pathogens and insect pests, increasing host susceptibility, and compromising plant immune responses (Son and Park, 2022; Roussin-Leveillee et al., 2024). Crops are therefore increasingly challenged by multiple stresses that occur simultaneously or sequentially, rather than by a single environmental constraint.

The development of crop varieties that can maintain stable performance under such conditions has become a major priority in modern agriculture. Conventional breeding has contributed greatly to improvements in yield, quality, and stress resistance, but the process remains time-consuming and heavily dependent on repeated field evaluation. Its limitations become particularly evident for complex traits controlled by many genes and strongly influenced by environmental conditions. Under these circumstances, selection based on a limited number of phenotypic observations or molecular markers may fail to capture the full complexity of genotype–phenotype–environment relationships (Wallace et al., 2018). Meanwhile, advances in high-throughput sequencing, imaging, remote sensing, omics technologies, and artificial intelligence (AI) have greatly expanded both the amount of data available to plant breeders and the ability to interpret it (Sarkar et al., 2024). Genomic, transcriptomic, metabolomic, phenomic, and environmental datasets can now be generated at unprecedented resolution. These resources offer new opportunities to describe crop performance more comprehensively, but they also present a considerable analytical challenge. The information is often high-dimensional, heterogeneous, and collected across different developmental stages, populations, and environments. Extracting useful breeding information from such data requires methods capable of identifying complex and often nonlinear relationships.

Digital breeding has emerged as a predictive and data-driven approach that integrates genomic, phenotypic, multi-omics, and environmental data using advanced computational and AI tools. It supports trait prediction, the selection of promising genotypes, and the design of breeding strategies while reducing reliance on subjective and labor-intensive selection processes. In practice, digital breeding can help estimate the performance of untested genotypes, identify promising parents and progeny, and compare breeding strategies before extensive field evaluation. It does not replace conventional breeding but rather extends it by enabling breeders to make better use of existing data and reduce the number of candidates requiring experimental evaluation.

AI has further broadened the scope of crop breeding. Machine learning (ML) is increasingly used to model complex relationships among genotype and phenotype, while deep learning (DL) has shown particular value for plant images, hyperspectral data, time-series measurements, and large-scale omics profiles (Crossa et al., 2025). These approaches are being applied to yield prediction, stress tolerance assessment, disease detection, phenotype classification, genomic prediction, and the prioritization of breeding materials. Their main advantage lies not simply in handling large datasets, but in combining different sources of information that are difficult to interpret separately. Despite this progress, several limitations continue to restrict the broader application of AI in crop breeding. Many studies are based on relatively small or unbalanced datasets, while variation in experimental design, phenotyping protocols, metadata description, and data formats limits comparison, integration, and reuse across studies (Papoutsoglou et al., 2020; van Dijk et al., 2021). Predictive performance may also decline when models are applied to genetically distinct populations or to environments, locations, and years that differ from those represented in the training data (Alemu et al., 2024). A further challenge is the limited interpretability of complex ML and DL models, which can make it difficult to assess whether their predictions reflect biologically meaningful relationships and thereby reduce confidence in their practical use (Mostafa et al., 2023). These concerns have encouraged the development of explainable modeling frameworks, standardized and reusable data resources, and approaches that incorporate prior biological knowledge into model construction and interpretation.

Rapid advances in AI are opening new opportunities across agriculture and drawing growing attention to their potential in crop improvement. Accordingly, we review how AI is being used to integrate and analyze large-scale genomic, phenotypic, multi-omics, and environmental data, how AI models support prediction and decision-making in crop breeding, and how they are being translated into practical digital breeding applications. Specifically, this review addresses three questions: what data foundations are required for AI-enabled crop breeding, which AI models are most appropriate for different breeding tasks, and what evidence is needed to determine whether these approaches improve breeding decisions and outcomes. Relevant literature was selected to provide representative, rather than exhaustive, coverage of AI methods and applications in crop breeding. Finally, this review discusses the future prospects and broader potential of AI-driven digital breeding.

## 2 From conventional and data-assisted breeding to AI-driven breeding

**Figure 1:**
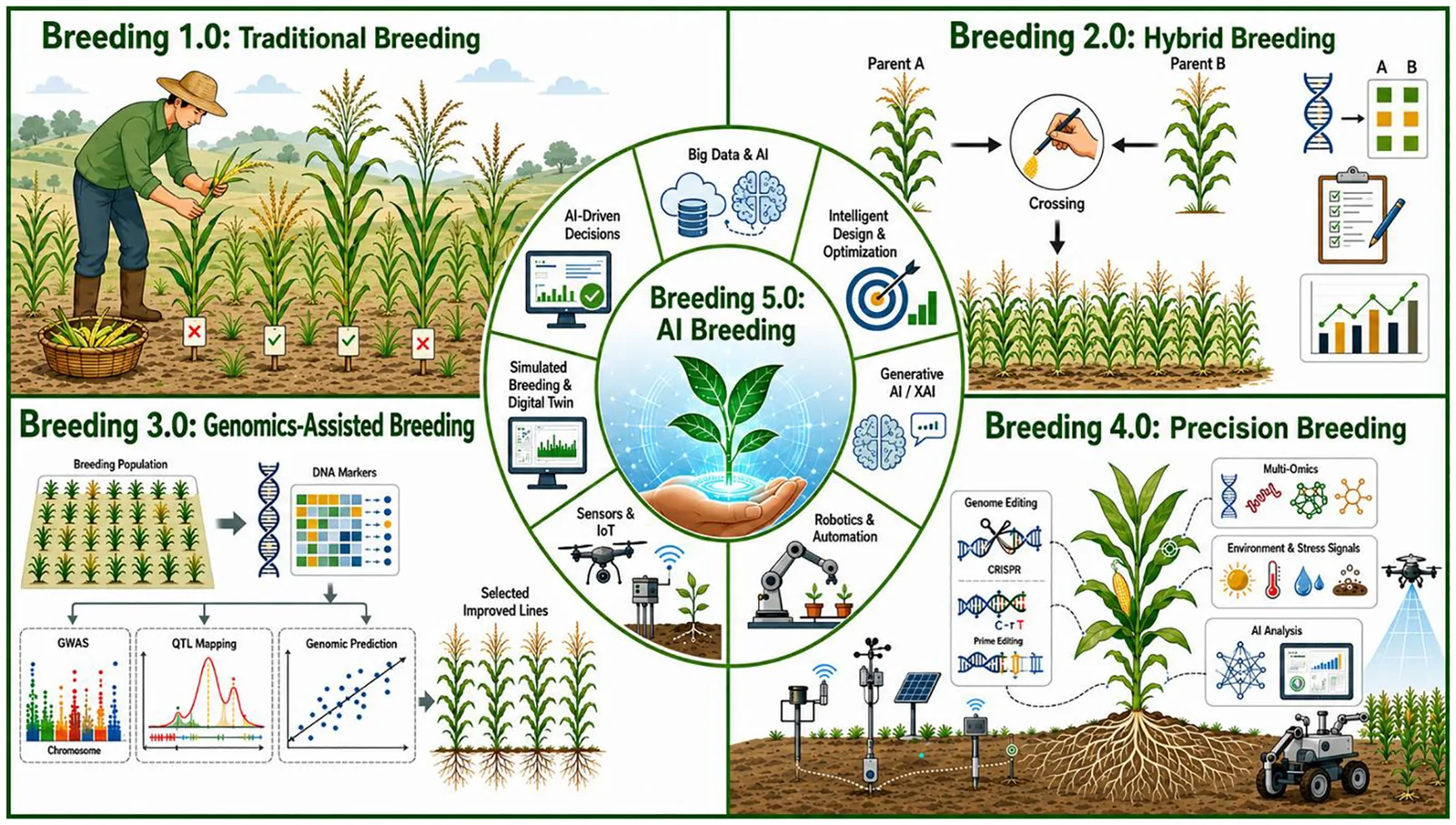
Evolution of crop breeding from Breeding 1.0 to Breeding 5.0. Breeding 1.0 is centered on visual assessment and phenotypic selection, whereas Breeding 2.0 introduces deliberate crossing, genetic principles, and statistical evaluation to exploit hybrid performance. Breeding 3.0, characterized as genomics-assisted breeding, extends selection through genome-wide markers, quantitative trait locus mapping, genome-wide association studies, and genomic prediction. Breeding 4.0 integrates genome editing, multi-omics, environmental sensing, high-throughput phenotyping, and AI-assisted analysis, in which AI serves as one component of a broader technology-enabled breeding workflow. Breeding 5.0, defined as AI breeding, places AI at the core of the breeding framework, using multimodal data to provide AI-driven decision support and coordinate digital twins, intelligent experimental design, robotics, automation, and sensor networks throughout the breeding cycle. These stages represent broad conceptual transitions rather than strictly separated periods; their technologies and practices overlap and continue to coexist in contemporary breeding programs.

Plant breeding aims to develop crop varieties with improved yield, quality, adaptation, and resistance to biotic and abiotic stresses. Over time, the methods used to achieve these goals have changed markedly, reflecting advances in genetics, biotechnology, phenotyping, and data analysis, and this evolution can be broadly described as Breeding 1.0–5.0 (Wallace et al., 2018; Fu et al., 2026). Although this framework does not represent a universally accepted or strictly sequential taxonomy, the evolution of crop breeding can be conceptualized as five broad stages (**Figure 1**), with the corresponding approaches often coexisting and overlapping in practice.

In Breeding 1.0, crop improvement relied primarily on the selection of superior plants from naturally occurring variation. Breeders assessed visible characteristics such as vigor, maturity, yield, quality, and stress tolerance and retained plants with desirable performance. This approach laid the foundation for modern crop breeding and contributed to the development of many locally adapted varieties. Its success, however, depended heavily on repeated field observation, environmental conditions, and the breeder’s experience. Because the genetic basis of most traits was largely unknown, improvement often required several generations of selection. The emergence of hybrid breeding marked the transition to Breeding 2.0. Controlled crossing allowed breeders to combine favorable traits from genetically distinct parents and to exploit heterosis for improved crop performance. Advances in genetics, experimental design, and statistics made crossing and selection more systematic, although field-based phenotypic evaluation remained central to the breeding process.

The introduction of molecular markers and genome-wide information led to Breeding 3.0. At this stage, breeders began to use marker–trait associations and genome-wide variation to identify promising materials before complete field evaluation (Varshney et al., 2005). Marker-assisted selection (MAS), quantitative trait locus (QTL) mapping, genome-wide association studies (GWAS), and genomic selection (GS) improved both the speed and accuracy of selection (Kumar et al., 2024). GS was particularly valuable for complex traits, as it enabled prediction based on marker effects distributed across the genome rather than on a small number of major loci. With the expansion of biotechnology and multi-layered biological data, plant breeding advanced to Breeding 4.0 (Wallace et al., 2018). Transgenic approaches and genome editing made it possible to modify target genes more directly, while transcriptomic, proteomic, metabolomic, and phenomic analyses provided a more detailed view of the processes underlying crop performance. These developments strengthened the link between gene function and agronomic traits and allowed crop improvement to become more targeted and precise.

The current shift toward Breeding 5.0 reflects a further change in the role of data and computation. AI is increasingly used to integrate genomic, phenomic, multi-omics, environmental, and historical breeding data for trait prediction, candidate selection, and breeding design. ML and DL can reveal patterns that are difficult to capture with conventional analytical methods, while explainable AI (XAI), generative AI, intelligent robotics, and simulated breeding are extending the scope of prediction, automation, and virtual evaluation. The distinction between Breeding 5.0 and other breeding frameworks lies not in whether computational tools contribute to decision-making, but in the degree to which they are integrated and automated across the breeding process. Predictive algorithms and optimization methods already contributed to breeder decision-making in earlier frameworks, whereas Breeding 5.0 places greater emphasis on the coordinated use of AI to predict, rank, and optimize breeding options across multiple stages. Even so, AI does not replace breeder expertise. Its value continues to depend on the quality of the underlying data, the biological relevance of the models, and validation under field conditions. AI-driven breeding is therefore better understood as an extension of the breeder’s capacity rather than a substitute for established breeding approaches.

## 3 Data foundations for digital breeding

Digital breeding integrates genomic, phenotypic, multi-omics, and environmental data across the breeding process and applies AI to guide trait prediction, early selection, parent selection, crossing design, and cultivar development, with the potential to improve breeding precision, shorten breeding cycles, and increase the efficiency of resource use. Digital breeding extends beyond genomic prediction, which relies mainly on genome-wide marker data to estimate breeding values or phenotypic performance, by incorporating a broader range of biological and environmental information across the breeding process (Crossa et al., 2017; Jeon et al., 2023).

The success of digital breeding depends largely on the quality, consistency, and connectivity of the underlying data. The data must not only be generated at sufficient scale but also curated, standardized, preprocessed, and integrated in ways that preserve their biological meaning and maintain reliable links across samples, environments, and developmental stages. This section describes the main data foundations of digital breeding, from germplasm resources and data generation to data preparation and integration.

### 3.1 Germplasm

Germplasm is the starting point of digital breeding because the range of traits and alleles available for prediction and selection is ultimately determined by the diversity of the materials being evaluated. Landraces, traditional varieties, modern cultivars, breeding lines, and crop wild relatives together capture a wide spectrum of genetic variation, including alleles that may have been lost or overlooked during domestication and modern breeding. Broad germplasm diversity therefore increases the likelihood of identifying useful genetic variation for agronomic traits and crop improvement (Jayakodi et al., 2025).

Nearly six million accessions of major crops, locally important crops, and crop wild relatives are maintained in more than 869 genebanks across 116 countries, underscoring the scale of the genetic resources available for crop improvement (Hanson et al., 2025). However, many collections remain difficult to use effectively because their genetic and phenotypic characteristics are incompletely documented and information is often dispersed across separate databases. Large-scale genomic characterization can help reveal useful variation and improve the connection between conserved germplasm and breeding programs (Mascher et al., 2019). Germplasm should therefore be viewed not simply as conserved biological material, but as a data-rich resource for identifying useful alleles, donor lines, and parental combinations.

### 3.2 Data generation for digital breeding

The performance of digital breeding depends on the quality, scale, and diversity of the data available for analysis. Genomic, phenotypic, and multi-omics data capture different layers of biological variation, from DNA sequence to observable traits and the molecular processes that connect them (Wang et al., 2024). Collecting these data across diverse genotypes and environments allows genotype-by-environment interactions to be considered and can improve the reliability of trait prediction (Jung et al., 2025).

#### 3.2.1 Genomic data generation

Plant genomic resources have expanded rapidly with advances in sequencing, genome assembly, and computing. Genome sequences are now available for more than 1,800 plant species. In 2024 alone, genome sequences were published for 500 species, including 370 that had not previously been sequenced (Schwacke et al., 2025). This expansion has moved plant genomics beyond major crops and model plants to encompass a much broader range of cultivated, wild, and previously understudied species.

Next-generation sequencing has also transformed population-level genomic analysis. High-throughput marker platforms now allow large numbers of loci to be surveyed across entire genomes and associated with phenotypic variation at a scale that was previously difficult to achieve. Methods such as genotyping-by-sequencing (GBS), genotyping by targeted sequencing (GBTS), restriction site-associated DNA sequencing (RAD-seq), and target capture provide scalable approaches for variant discovery and genome-wide genotyping in plant breeding (Li et al., 2026b). Low-coverage sequencing combined with genotype imputation can further reduce the amount of sequence data required per individual, making large germplasm collections and breeding populations more practical to analyze (Long et al., 2022).

The accompanying growth in data volume has made computing capacity equally important. High-performance computing allows read alignment, genome assembly, variant calling, and population-level analyses to be distributed across multiple processors, substantially shortening the time required to process large datasets (Olbrich et al., 2024). For instance, an optimized pipeline running on the Rural Development Administration supercomputer completed whole-genome variant analysis of 849 pepper (*Capsicum*) accessions in 6.7 days, whereas the variant-calling step alone was estimated to require approximately 716 days on a conventional laboratory server (Lee et al., 2024). This study illustrates how parallel computing can bring population-scale genomic analysis within a practical breeding timescale.

Advances in sequencing technology have also improved plant genome analysis. Short-read sequencing is well suited to large-scale genotyping, whereas long-read sequencing provides a more complete view of genome structure. Because long reads can span repetitive and structurally complex regions, they improve assembly continuity and reveal sequence and structural variants that are often missed by short-read approaches (Pucker et al., 2022). Together with scalable genotyping methods and high-performance computing, these technologies have enabled more detailed characterization of genetic variation across larger and more diverse breeding populations.

#### 3.2.2 Phenomic data generation

**Figure 2:**
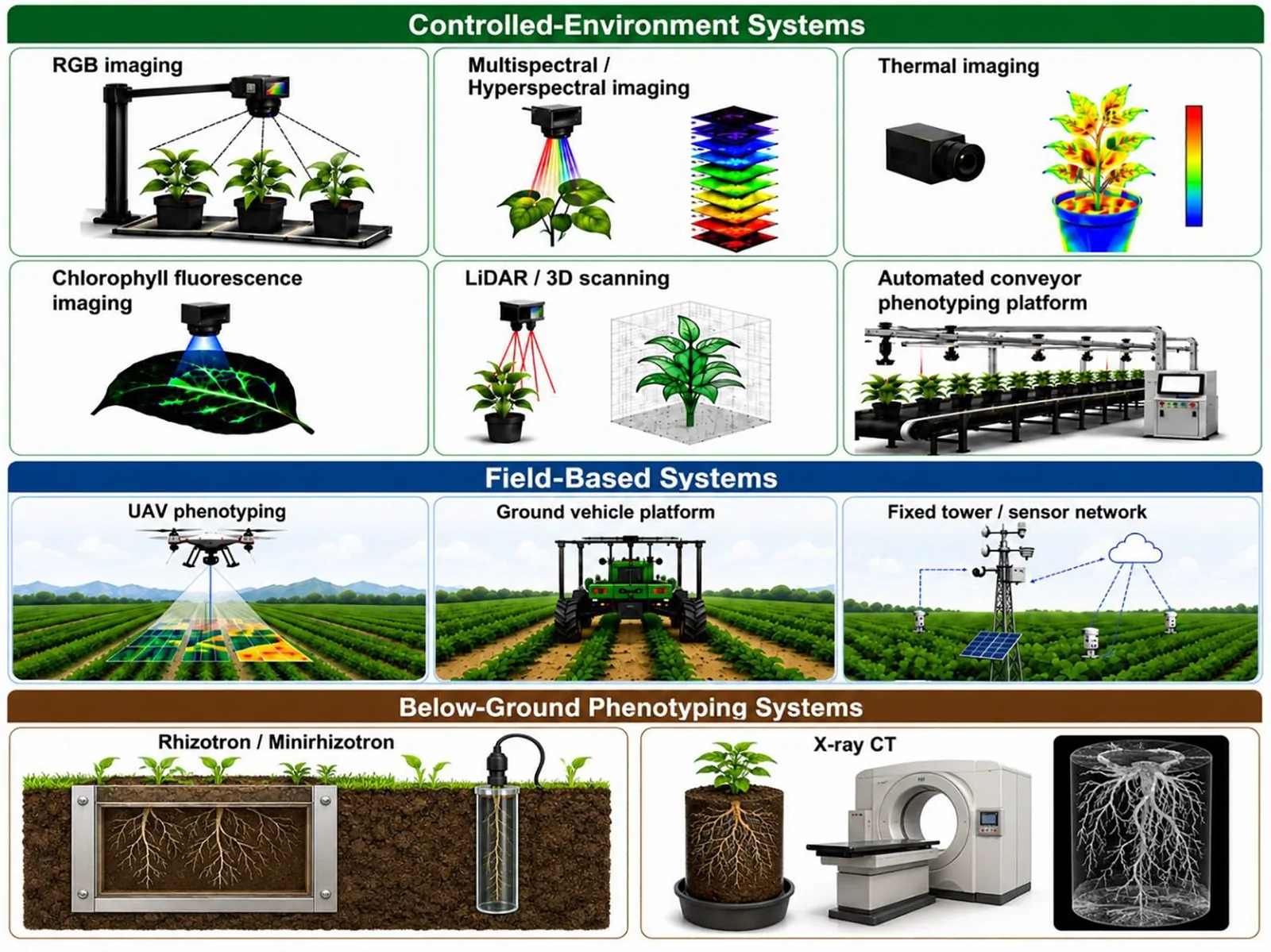
Major phenotyping systems used in crop breeding. Controlled-environment platforms include red-green-blue (RGB) imaging for quantifying plant size, morphology, color, and growth; multispectral and hyperspectral imaging for assessing pigments, nutrient status, and stress-related spectral responses; thermal imaging for detecting canopy temperature and water-related stress; chlorophyll fluorescence imaging for evaluating photosynthetic performance; light detection and ranging (LiDAR) and three-dimensional scanning for reconstructing plant architecture and estimating structural traits; and automated conveyor systems for repeated, high-throughput measurements under standardized conditions. Field-based platforms include unmanned aerial vehicles for rapid assessment of crop growth, stress, and disease over large areas; ground vehicle systems for high-resolution measurements at the plot level; and fixed towers and sensor networks for continuous monitoring of crop and environmental conditions. Below-ground phenotyping systems include rhizotrons and minirhizotrons for repeated observation of root growth in soil, and X-ray computed tomography for non-destructive three-dimensional reconstruction of root system architecture.

Phenotypic data provide the observable link between genetic variation and agronomic performance and are therefore central to digital breeding. Image-based high-throughput phenotyping has expanded the scale and efficiency of plant trait measurement through advances in imaging and computational analysis (Murphy et al., 2024). Under controlled conditions, a range of imaging technologies can be used to capture structural and physiological traits rapidly and with minimal disturbance to the plant (**Figure 2**). Red-green-blue (RGB) imaging provides standard color images from which plant size, projected leaf area, shape, color, and visible stress symptoms can be quantified (Kior et al., 2024). Multispectral imaging records reflectance in selected wavelength bands and is commonly used to derive vegetation indices associated with plant biomass, pigment status, and overall vigor (Zhang et al., 2024). Hyperspectral imaging extends this approach across hundreds of narrow, contiguous bands, providing finer information on biochemical composition, water and nutrient status, and early stress responses (Zhang et al., 2024). Thermal imaging measures plant surface temperature and can indicate changes in transpiration, stomatal conductance, and water stress (Walsh et al., 2024), whereas chlorophyll fluorescence imaging provides information on photosynthetic performance and stress-induced impairment of the photosynthetic apparatus (Park et al., 2024). Three-dimensional imaging, using stereo vision, depth cameras, laser scanning, or related approaches, enables detailed measurement of plant architecture, height, volume, leaf angle, and spatial organization beyond what can be captured from two-dimensional images alone (Li et al., 2025). Automated conveyor-based systems transport plants to fixed imaging stations, whereas sensor-to-plant platforms move cameras and sensors around stationary plants. Both approaches allow the same individuals to be measured repeatedly under standardized conditions, generating time-series data on growth, development, and treatment responses (Li et al., 2021; Poorter et al., 2023). Repeated, non-destructive measurements in controlled-environment phenotyping systems can reveal subtle or transient phenotypic differences that may be missed by conventional endpoint measurements (Poorter et al., 2023). However, phenotypes measured under controlled conditions do not always translate directly to field performance, and therefore require validation under realistic environments.

Field-based phenotyping extends plant measurement to larger breeding populations under realistic and spatially variable conditions (**Figure 2**). Unmanned aerial vehicles (UAVs), tractor- or vehicle-mounted platforms, fixed sensor networks, and field robots can repeatedly assess canopy height, cover, biomass, temperature, vigor, disease symptoms, and stress responses at plot or field scale. UAVs are particularly effective for rapid surveys over large areas (Tanaka et al., 2024), whereas ground-based platforms generally provide closer-range measurements at higher spatial resolution (Xu and Li, 2022). Fixed sensor networks provide continuous measurements of weather, soil, and crop conditions, helping phenotypic variation to be interpreted in relation to spatial field heterogeneity and changing environmental conditions (Soussi et al., 2024).

Below-ground phenotyping represents a distinct component of plant phenotyping because root traits require specialized measurement approaches that differ from those used for above-ground field observations (**Figure 2**). Rhizotrons and minirhizotrons allow root growth to be followed repeatedly in soil (Chandnani and Soolanayakanahally, 2025), while X-ray computed tomography can reconstruct root systems in three dimensions without destroying the sample (Herrero-Huerta et al., 2022; van der Bom et al., 2025). These methods can reveal root architecture, depth distribution, branching patterns, and responses to water or nutrient limitation, although their throughput remains lower than that of above-ground imaging systems.

Across controlled-environment, field-based, and below-ground phenotyping platforms, image and sensor data must first be translated into robust and reproducible quantitative traits before they can support genetic analysis and selection. Reliable trait extraction depends on careful calibration, standardized measurement protocols, comprehensive metadata, and appropriate validation, because factors such as illumination, weather, soil background, developmental stage, and sensor configuration can all influence the resulting measurements. Once these sources of variation are adequately accounted for, high-throughput phenotyping can generate the phenomic data required for trait prediction, genotype-by-environment analysis, and the identification of superior breeding lines.

#### 3.2.3 Functional omics data generation

**Figure 3:**
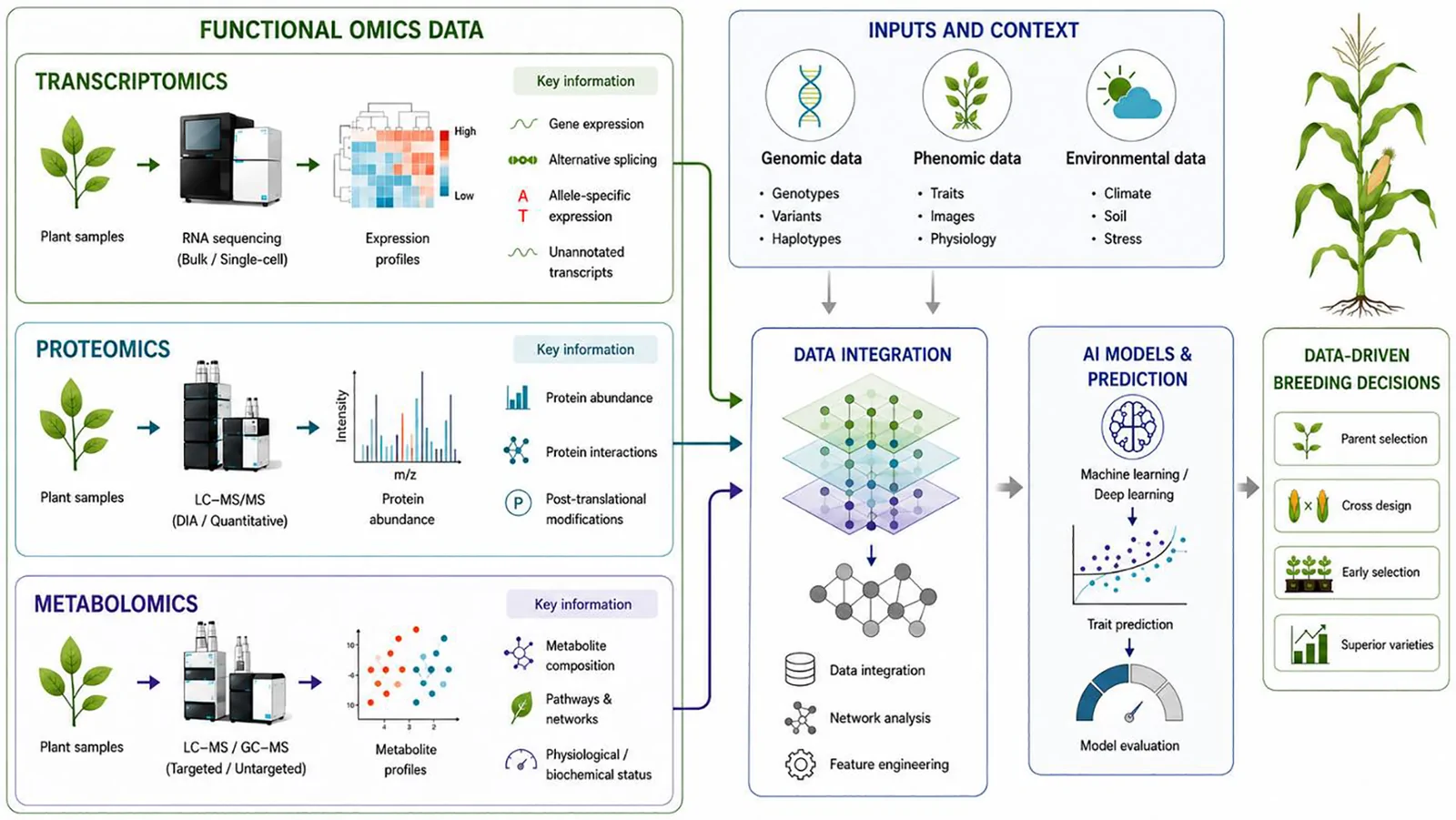
Functional omics data and their contributions to digital breeding. Transcriptomics characterizes gene activity through bulk and single-cell RNA sequencing, revealing gene expression, alternative splicing, allele-specific expression, and previously unannotated transcripts. Proteomics captures protein abundance, molecular interactions, and post-translational modifications using liquid chromatography–tandem mass spectrometry and quantitative proteomic approaches. Metabolomics profiles metabolite composition and physiological or biochemical status through targeted and untargeted analyses based on gas or liquid chromatography–mass spectrometry. These functional omics data are integrated with genomic data, phenotypic data, and environmental information, which provide additional inputs and contextual information for data integration and AI-based prediction. Genomic data provide information on genotypes, variants, and haplotypes; phenotypic data describe traits, images, and physiological responses; and environmental data capture factors such as climate, soil conditions, and stress exposure. Collectively, these molecular, genomic, phenotypic, and environmental data enable trait prediction and data-driven breeding decisions. This conceptual figure was created with the assistance of generative AI and subsequently reviewed and revised by the authors for scientific accuracy.

Functional omics data, including transcriptomic, proteomic, and metabolomic profiles, provide dynamic molecular information that complements genomic variation in digital breeding (**Figure 3**). Genomic data define the inherited potential of breeding materials, but they do not fully explain how that potential is expressed under different developmental and environmental conditions (Wang et al., 2024). Gene expression, protein abundance and modification, and metabolite composition help capture the molecular processes that connect genotype, environment, and phenotype (Zhang et al., 2022b; Zhang et al., 2025). Integrated with genomic, phenotypic, and environmental data, these layers can support a more functional interpretation of genetic variation, strengthen the prediction of complex traits, and guide breeding decisions. Although epigenomic, ionomic, and microbiome data can also contribute to digital breeding, they are not discussed here because this section focuses on the functional omics layers most commonly integrated with genomic and phenotypic data in current AI-assisted breeding studies.

##### 3.2.3.1 Transcriptomic data generation and resources

Advances in RNA sequencing (RNA-seq), reference genome resources, and computational analysis have greatly expanded the range and scale of plant transcriptomic data. Bulk RNA-seq is now routinely used to examine gene expression across tissues, developmental stages, genotypes, and environmental conditions. Beyond measuring transcript abundance, these datasets can reveal differential expression, alternative splicing, allele-specific expression, and previously unannotated transcripts, providing a dynamic view of gene activity that complements genomic information (Upton et al., 2023). Publicly available plant transcriptomic resources have expanded markedly in both scale and resolution. The 1000 Plant Transcriptomes Initiative generated transcriptome data from 1,342 samples representing 1,173 green plant species, extending coverage far beyond major crops and model plants (Carpenter et al., 2019). PlantExp brings together 131,423 uniformly processed public RNA-seq samples from 85 species across 24 plant orders and supports analyses of gene expression, alternative splicing, co-expression, and cross-species conservation (Liu et al., 2023). Complementing these large public collections, the JGI Plant Gene Atlas comprises 2,090 tissue samples from 18 species generated under standardized conditions across diverse tissues, developmental stages, growth environments, and abiotic stress treatments (Sreedasyam et al., 2023). Transcriptomic resources have also begun to move beyond bulk tissue profiles. scPlantDB, for example, integrates approximately 2.5 million cells from 67 single-cell datasets across 17 plant species, together with standardized expression profiles and cell-type annotations (He et al., 2024). These resources reflect not only the rapid accumulation of plant transcriptomic data, but also a broader shift toward datasets that are more comparable, reusable, and informative across species, tissues, environments, and cell types.

##### 3.2.3.2 Proteomic data generation and resources

Plant proteomics has advanced rapidly with improvements in mass spectrometry and quantitative analysis. Liquid chromatography–tandem mass spectrometry (LC–MS/MS) is now widely used to profile protein abundance across tissues, developmental stages, genotypes, and environmental conditions. Data-independent acquisition and label-free or isotope-based quantification have further improved the consistency and comparability of large-scale datasets, while specialized workflows enable the analysis of protein interactions and post-translational modifications (Yan et al., 2022). The most comprehensive plant proteome resources were initially developed for the model plant *Arabidopsis thaliana*, notably ATHENA and the Arabidopsis PeptideAtlas (Mergner et al., 2020; van Wijk et al., 2021). These resources provide broad tissue coverage and consistently processed reference data, whereas comparable resources remain less complete for crop species. In rice (*Oryza sativa*), the Rice PeptideAtlas compiles publicly available mass spectrometry datasets reprocessed through a uniform analytical pipeline. In a complementary study, the uniform reanalysis of public rice phosphoproteomic datasets identified 15,565 phosphorylation sites and related this information to sequence variation in the 3,000 Rice Genomes Project (Ramsbottom et al., 2024). In maize (*Zea mays*), the Maize PeptideAtlas reanalyzes publicly available raw tandem mass spectrometry data from diverse genetic backgrounds, sample types, and experimental conditions through a uniform processing and metadata annotation pipeline (van Wijk et al., 2024). In soybean (*Glycine max*), the Soybean Proteome Database is an earlier crop-specific resource that compiles two-dimensional gel electrophoresis-based and gel-free proteomics data from several organs, tissues, and subcellular compartments (Ohyanagi et al., 2012). However, much of its content was generated from a single cultivar and a limited range of developmental and stress conditions. qPTMplants integrates experimentally identified and quantitatively profiled post-translational modifications across 43 plant species, 23 modification types, and 583 experimental conditions (Xue et al., 2022). Crop proteomic datasets are also deposited in the PRoteomics IDEntifications Database, which preserves raw mass spectrometry files, peptide and protein identification results, and associated metadata for reuse and reanalysis (Perez-Riverol et al., 2025). Despite these advances, crop proteomic resources remain uneven in their representation of genetic diversity and experimental conditions, and population-scale datasets developed specifically for breeding applications remain limited.

##### 3.2.3.3 Metabolomic data generation and resources

Plant metabolomics is based largely on mass spectrometry coupled with chromatographic separation. Gas chromatography–mass spectrometry is commonly used for volatile compounds and derivatized primary metabolites, whereas liquid chromatography–mass spectrometry provides broader coverage of chemically diverse and specialized metabolites. Targeted approaches quantify predefined compounds, while untargeted analyses survey a wider range of metabolic features across biological samples (Alseekh et al., 2021; Martinez-Rivas and Fernie, 2024). A growing number of plant metabolome resources now support data reuse and metabolite identification. RefMetaPlant provides reference metabolomes for 153 plant species and includes more than one million experimentally generated mass spectra (Shi et al., 2024). The RIKEN Plant Metabolome MetaDatabase brings together plant mass-spectrometry data with raw files, spectra, metabolite annotations, and detailed experimental metadata (Fukushima et al., 2022). PMhub serves a complementary role by integrating chemical, spectral, pathway, and genetic information for 188,837 plant metabolites, together with more than 1.4 million standard or *in silico* high-resolution tandem mass spectrometry (HRMS/MS) spectra (Tian et al., 2024). Broader repositories such as MetaboLights and Metabolomics Workbench also preserve raw and processed metabolomics data and associated metadata across species and analytical platforms (Sud et al., 2016; Yurekten et al., 2024). Moreover, complementary resources support metabolic prediction and pathway-level interpretation. The Plant Comparative Metabolome Database provides genome-based predicted metabolic profiles for 530 plant species (Hu et al., 2024), while the Plant Metabolic Network integrates enzymes, reactions, metabolites, and pathways across 155 plant and green algal genomes (Hawkins et al., 2025). Despite their growing availability, standardized population-scale metabolomic resources designed for crop breeding remain limited.

##### 3.2.3.4 Predictive value and practical limitations of functional omics

The expansion of functional omics resources does not necessarily translate into greater value for digital breeding. Their practical utility depends on whether they provide predictive information beyond genomic markers and whether any improvement is sufficient to justify the associated sampling burden, missingness, lower throughput, and biological specificity. Among the three layers, transcriptomic data are currently the most mature and have been evaluated most extensively for trait prediction. Transcriptomic profiles capture condition-dependent gene activity and can therefore complement genomic variation, although their contribution varies substantially with the trait, population, and prediction setting. In a maize hybrid breeding population, mRNA profiles measured in the parental lines were the strongest single predictors of hybrid grain yield, whereas genomic data performed best for grain dry matter content (Schrag et al., 2018). Combining genomic and transcriptomic information provided consistently high predictive ability across both traits. In a diverse panel of maize inbred lines, seedling transcriptomes also supported the prediction of adult flowering time, plant height, and grain yield beyond models based only on population structure (Azodi et al., 2020). Although genetic marker-based models consistently outperformed transcript-based models and simply combining all transcript and marker features provided little additional benefit, prediction performance improved when the most informative features identified by the separate models were integrated. These studies suggest that transcriptomic data can provide useful complementary information, but that their marginal value is conditional rather than general. Transcript abundance is also highly dependent on tissue, developmental stage, sampling time, and environment. Generating comparable transcriptomic profiles across large breeding populations and multiple environments therefore requires repeated, carefully standardized sampling, whereas genomic markers can generally be measured once for each line.

Proteomic data offer a closer view of cellular function by capturing variation in protein abundance, turnover, regulation, and post-translational modification. They may therefore reveal biological differences that cannot be inferred directly from transcript levels. In a maize association panel, protein and transcript abundance showed only limited concordance, and many protein quantitative trait loci had no corresponding expression quantitative trait loci, pointing to extensive post-transcriptional regulation (Jiang et al., 2019). Their use in breeding prediction, however, remains constrained by demanding sample preparation, incomplete proteome coverage, limited analytical throughput, and frequent missing values. Plant proteome studies have shown that extraction and digestion procedures can substantially affect the number of peptides and proteins recovered, underscoring the need for standardized workflows (Song et al., 2018). Missing values are also common in mass-spectrometry-based quantitative proteomics and can influence downstream analyses unless they are handled carefully (Wang et al., 2020). Differences in sample preparation, instrumentation, analytical depth, and data-processing pipelines further limit comparability across studies. Although proteomic variation has been characterized in crop diversity panels, evidence that proteomic data provide consistent gains over genomic prediction in large operational breeding populations remains scarce.

Metabolomic profiles provide an even more immediate view of plant physiological and biochemical status because they integrate the downstream effects of genotype, development, and environment. Their predictive value may therefore be particularly high for traits closely related to composition or physiological performance. In barley (*Hordeum vulgare*), the addition of metabolomic information substantially improved the prediction of breeding values for lines without phenotypic records, whereas little further benefit was observed when phenotypic records for those lines were already available (Guo et al., 2023). This result illustrates both the promise and the practical limitation of metabolomic prediction. Metabolite profiles may strengthen early selection, but collecting them for large numbers of candidates reduces the throughput advantage normally associated with prediction without direct phenotyping. Metabolomic data are also highly sensitive to tissue, developmental stage, sampling time, sample handling, and short-term environmental variation (Alseekh et al., 2021). Differences among analytical platforms, incomplete metabolite identification, the widespread use of relative rather than absolute quantification, and inconsistent annotation further hinder comparison and integration across studies.

Overall, evidence from representative crop breeding populations remains far more limited than that from diversity panels, selected germplasm, and controlled experiments. Existing studies show that transcriptomic and metabolomic data can improve prediction for particular traits and validation settings, but they do not yet demonstrate a consistent advantage across populations, environments, and breeding cycles (Schrag et al., 2018; Azodi et al., 2020; Guo et al., 2023). Progress will therefore require more than simply expanding data resources. Each omics layer must be evaluated directly against genomic prediction under realistic breeding conditions. Future studies should combine standardized, replicated sampling across crop species, breeding populations, tissues, developmental stages, and environments with explicit tests of whether the added predictive gain is sufficient to offset the associated sampling burden, missingness, lower throughput, and temporal and tissue specificity.

#### 3.2.4 Environmental data generation

Environmental data are needed to understand how genetic differences are translated into phenotypic variation across locations and growing conditions (Napier et al., 2023). Weather stations, soil sensors, and wireless monitoring networks can capture temperature, rainfall, humidity, solar radiation, wind, soil moisture, and soil temperature directly at breeding sites (Soussi et al., 2024). These site-specific observations can be complemented by satellite-derived and gridded environmental datasets, which extend coverage across locations and growing seasons (Sishodia et al., 2020).

Several public resources can be used to characterize growing environments. NASA Prediction of Worldwide Energy Resources provides global time-series data on solar radiation and meteorological conditions derived from satellite-based products and atmospheric reanalysis models (Sparks, 2018). ERA5-Land provides hourly estimates of land-surface and meteorological variables from 1950 onward at a spatial resolution of approximately 9 km (Muñoz-Sabater et al., 2021). SoilGrids provides global predictions of soil properties, including organic carbon, total nitrogen, pH, cation-exchange capacity, bulk density, and sand, silt, and clay contents, at 250-m resolution across six standard depth intervals (Poggio et al., 2021). These resources are useful where local observations are incomplete, although their gridded and model-based estimates may not capture fine-scale variation within individual breeding fields. Integrating such environmental information with breeding records requires data platforms that can link trials and locations with germplasm, phenotypic, and genotypic data. BreedBase, for example, supports the management of germplasm, field trials, phenotypic and genotypic data, and data exchange through the Breeding Application Programming Interface (Morales et al., 2022). However, environmental information is often obtained from heterogeneous and separately managed data sources, making its consistent integration with breeding trials difficult (Brown et al., 2020).

For digital breeding, environmental data are essential for characterizing genotype-by-environment interactions and predicting crop performance across locations and seasons. Their usefulness depends on accurate alignment with genotype, plot, developmental stage, and measurement period. Differences in spatial and temporal resolution, sensor calibration, variable definition, and management metadata still limit comparisons among trials. More consistent field-level monitoring and standardized environmental records will therefore be needed to support reliable multi-environment prediction and breeding decisions.

### 3.3 Data integration

Building on previous reviews of the principles and computational tools used to integrate multi-omics, phenotypic, and environmental data (Jamil et al., 2020; Cembrowska-Lech et al., 2023; Baiao et al., 2025; Morabito et al., 2025), this section highlights the preprocessing steps, integration strategies, and analytical considerations most relevant to digital breeding.

In digital breeding, diverse datasets must be carefully prepared before they can be analyzed jointly using statistical or AI-based models. For practical purposes, this preparation can be organized into four key steps: data harmonization, quality control, normalization and scaling, and feature extraction and selection. Data harmonization establishes reliable links among records from different sources by aligning identifiers, data formats, measurement units, and metadata (Deng et al., 2023; Saras et al., 2025). Consistent identifiers are therefore needed for germplasm, genotypes, plots, trials, locations, years, treatments, tissues, developmental stages, and sampling times. This is particularly important in breeding datasets because measurements from the same genotype may originate from different plots, environments, developmental stages, or experimental treatments. Trait definitions, measurement units, data formats, ontologies, and metadata should therefore be standardized to reduce the risk of incorrect sample matching and spurious associations (Deng et al., 2023). Environmental and management records should also be aligned spatially and temporally with the corresponding trials and, where relevant, with specific developmental stages or sampling periods (Xu, 2016).

Quality control assesses data for missing, erroneous, unreliable, or technically compromised observations before integration. Genomic data are commonly filtered according to marker quality, missingness, minor allele frequency, and, where relevant, unexpected heterozygosity, with thresholds selected according to the crop and population structure (Pavan et al., 2020). Phenotypic records should be examined for missing values, outliers, measurement reliability, inconsistencies in the experimental design, and spatial variation within the field, although genuine biological extremes should not be removed without further evaluation. High-throughput phenotyping data additionally require sensor calibration and assessment of image or signal quality, with attention to variation caused by illumination, viewing geometry, platform operation, and measurement timing (Li et al., 2014; Poorter et al., 2023). Omics datasets should likewise be evaluated for low-quality measurements, technical artifacts, and batch effects, whereas environmental records require checks for missing observations, sensor malfunction, temporal inconsistencies, and physically or climatologically implausible values (Dunkerly et al., 2024; Yu et al., 2024).

Normalization, statistical adjustment, and missing-data handling prepare records from different sources for joint analysis. RNA-seq count data generally require normalization to account for differences in sequencing depth and RNA composition across samples (Love et al., 2014), whereas proteomic and metabolomic measurements may require intensity normalization, transformation, scaling, and assessment or correction of technical and batch effects (Sun and Xia, 2024; Yu et al., 2024). Continuous predictors may be centered and scaled, strongly skewed variables transformed, and categorical predictors encoded in a form appropriate for the selected analytical model. Phenotypic observations from field trials may be adjusted for experimental design, environmental effects, and spatial heterogeneity using linear or mixed models, with genotype performance summarized as best linear unbiased estimates (BLUEs) when genotypic effects are treated as fixed or as best linear unbiased predictions (BLUPs) when they are treated as random (Bernal-Vasquez et al., 2014; Oakey et al., 2016). Missing data should be handled according to their cause, extent, and pattern. Sporadic missing values may be imputed using appropriate statistical or model-based methods, whereas block-wise missingness, in which an entire data layer is unavailable for some samples, may require integration methods that explicitly accommodate partially observed samples or incomplete overlap among data layers (Argelaguet et al., 2020; Flores et al., 2023).

Feature extraction and selection help condense complex datasets into variables that are more suitable for predictive modeling. Image and sensor data can be used to derive quantitative traits such as plant height, canopy cover, vegetation indices, canopy architecture, and growth dynamics (Mochida et al., 2019). In genomic and omics datasets, dimensionality can be reduced by removing low-quality or redundant variables, selecting informative features through sparse regularization, or summarizing correlated measurements as latent factors (Wang et al., 2021; Velten et al., 2022). Environmental data can similarly be summarized over defined developmental stages into biologically meaningful covariates, including growing degree days, cumulative solar radiation, precipitation, heat exposure, vapor-pressure deficit, evapotranspiration, and water-balance indices (Westhues et al., 2021). When carefully designed and validated, these steps can reduce redundancy and computational demand while preserving information relevant to phenotypic variation and breeding objectives. For model benchmarking, however, all imputation, normalization, batch-correction, and phenotype-adjustment parameters, as well as feature-selection decisions, should be estimated within each training fold and then applied to the corresponding validation data to prevent information leakage and overly optimistic performance estimates.

Once preprocessing is complete, data can be integrated at early, intermediate, or late stages (Baiao et al., 2025). Early integration combines processed variables from different data layers into a single feature matrix. Although simple to implement, this approach can be affected by differences in scale, sparsity, dimensionality, and the relative number of variables contributed by each layer. Intermediate integration combines data through shared or complementary representations, with individual methods differing in their analytical purpose. Unsupervised integration methods identify latent structures or unified representations without using outcome information. MOFA+, implemented in the MOFA2 package, uses unsupervised factor analysis to capture major sources of variation that are shared across or specific to individual data layers (Argelaguet et al., 2020). Similarity Network Fusion constructs a sample-similarity network for each data type and iteratively merges these networks into a unified representation for downstream analysis (Wang et al., 2014). Weighted gene co-expression network analysis (WGCNA) serves a distinct, primarily exploratory role by grouping correlated genes into co-expression modules and summarizing each module by its eigengene (Langfelder and Horvath, 2008). These module-level summaries can subsequently be associated with phenotypic or environmental traits or incorporated into downstream integrative analyses. Supervised integration methods use outcome information to identify coordinated features across data layers. The mixOmics framework supports data exploration, dimension reduction, feature selection, and the integration of multiple datasets, whereas its supervised Data Integration Analysis for Biomarker discovery using Latent cOmponents (DIABLO) method identifies correlated features across data layers that distinguish predefined phenotypic groups (Rohart et al., 2017; Singh et al., 2019). In late integration, also referred to as decision-level fusion, each data layer is analyzed separately, and the resulting model outputs or predictions are combined at the decision stage, allowing each layer to be processed and modeled according to its own characteristics (Baiao et al., 2025).

Overall, effective data integration relies not only on the analytical method but also on consistent identifiers, complete metadata, appropriate preprocessing, and careful alignment across data layers. Collecting genomic, phenotypic, omics, environmental, and management data from the same breeding populations across environments and developmental stages provides a foundation for reliable trait prediction and informed breeding decisions. The range of AI approaches used to analyze these integrated datasets is discussed in the following sections.

## 4 AI models and applications in crop breeding

The term AI was introduced in the 1955 proposal for the Dartmouth Summer Research Project, which helped establish AI as a distinct research field (McCarthy et al., 2006). Early AI relied largely on symbolic reasoning and rule-based problem solving, but progress was constrained by limited computing power, scarce digital data, and difficulties in representing complex knowledge. Subsequent advances in ML, together with the rapid growth of digital data and computing power, accelerated the development of modern AI. The success of AlexNet in 2012 further demonstrated the scalability of DL using graphics processing units (GPUs) (Krizhevsky et al., 2012). More recently, generative and agentic AI systems have expanded AI beyond individual predictions to content generation, tool use, and multi-step decision support. These developments have also expanded the range of AI models and applications available for plant breeding (**Table 1**).

**Table 1:** Major AI approaches used in crop breeding research.

AI category	Representative model	Core principle	Key strength	Major applications
Statistical and probabilistic models	RR-BLUP	Shrinks all marker effects toward zero using a common penalty	Stable with high-dimensional marker data	Genomic prediction and marker-effect estimation
	GBLUP	Predicts breeding values using a genomic relationship matrix	Efficient for highly polygenic traits	Genomic prediction of complex traits
	Bayesian regression	Estimates marker effects using model-specific prior distributions	Accommodates diverse marker-effect distributions	Genomic prediction across genetic architectures
	Bayesian network	Models conditional dependencies using a directed graph	Integrates prior knowledge and uncertainty	Regulatory network and trait prediction
	HMM	Infers latent states from sequential observations	Handles ordered genomic data effectively	Haplotype inference and genotype imputation
Machine learning	RF	Combines predictions from randomized decision trees	Captures nonlinear effects and ranks features	Genomic prediction and feature prioritization
	SVM	Performs maximum-margin classification or regression	Effective in high-dimensional datasets	Trait prediction and stress classification
	XGBoost	Builds a regularized ensemble of boosted decision trees	Models complex nonlinear patterns efficiently	Yield, quality, and genomic prediction
Deep learning	CNN	Learns spatial features using convolutional filters	Excels at image-based feature extraction	Disease detection and image phenotyping
	RNN	Models sequential and temporal dependencies	Handles time-series and sequence data	Time-series phenotyping and growth forecasting
	Transformer	Uses self-attention to model relationships across inputs	Captures long-range dependencies in parallel	Genomic, sequence, and multimodal prediction
Explainable AI	SHAP	Quantifies feature contributions using Shapley values	Provides local and global feature attribution	Identification of influential genes and markers
	Grad-CAM	Highlights predictive regions in convolution-based models	Provides intuitive spatial explanations	Localization of disease and stress symptoms
Generative AI	GAN	Trains a generator and discriminator adversarially	Generates realistic synthetic samples	Image synthesis and data augmentation
	VAE	Learns a probabilistic latent representation	Supports representation learning and generation	Omics representation and synthetic data generation
	LLM	Generates text by predicting token sequences	Synthesizes and organizes textual information	Literature mining, knowledge synthesis, and breeding decision support

### 4.1 Classical statistical and probabilistic models

Statistical and probabilistic models have long formed the principal analytical basis for crop prediction and data-informed breeding decisions. Phenotypic linear mixed models are widely used to account for experimental design, environmental and spatial variation, and genotype-by-environment interactions in single- and multi-environment trials (Bernal-Vasquez et al., 2014; Oakey et al., 2016). Genomic prediction extended these frameworks by incorporating genome-wide marker information (Meuwissen et al., 2001). It can be formulated by estimating marker effects directly, as in ridge-regression best linear unbiased prediction (RR-BLUP), or by predicting genomic breeding values from a marker-derived genomic relationship matrix, as in genomic best linear unbiased prediction (GBLUP) (VanRaden, 2008; Endelman, 2011). Bayesian genomic prediction methods, collectively known as the Bayesian alphabet, differ in the prior distributions and shrinkage or variable-selection assumptions assigned to marker effects (Meuwissen et al., 2001; Gianola et al., 2009). Multi-trait models exploit genetic correlations among traits, whereas multi-environment models account for genotype-by-environment interactions across testing environments (Burgueño et al., 2012; Jia and Jannink, 2012). Reaction-norm models further incorporate environmental covariates to describe variation in genotype performance along environmental gradients (Jarquin et al., 2014). These approaches remain important reference points and benchmarks for evaluating whether more complex AI models offer meaningful gains in predictive accuracy, generalization, or breeding utility.

Probabilistic graphical and state-space models provide complementary means of representing conditional dependencies and hidden biological structure. Bayesian networks represent conditional dependencies among variables through a directed graphical structure. In plant breeding, they have been used to investigate relationships among genomic and phenotypic traits and to predict crop performance from observed data (Yu et al., 2019; Dos Santos et al., 2020). Hidden Markov models (HMMs) are well suited to data with temporal or sequential structure. They infer a sequence of unobserved states from repeated measurements whose patterns change over time (McClintock et al., 2020). In plant research, HMMs have been applied to time-series remote-sensing images, phenological observations, and physiological signals to estimate crop developmental stages, classify crop types, and distinguish changes in plant stress status (Siachalou et al., 2015). They are therefore useful when observable measurements provide only indirect information about the underlying biological state of a plant or crop.

### 4.2 ML and DL models

Recent reviews have comprehensively summarized ML and DL models and their applications in crop breeding (Paul et al., 2025; Zhu et al., 2025; Tsega and Mullualem, 2026). Accordingly, here we outline the key differences between the two approaches and their representative algorithms.

ML is a branch of AI in which algorithms learn predictive relationships from predefined input variables. ML models generally rely on structured data and often involve manual feature selection or engineering, whereas DL can learn feature representations directly from raw data. For small, structured datasets, conventional ML often has lower data and computational demands than DL, although their relative performance depends on model architecture, regularization, trait architecture, sample structure, and validation design. In digital breeding, ML has been applied to trait and yield prediction, stress-response classification, and the selection of promising genotypes. Among ML algorithms, random forest (RF), support vector machine (SVM), and extreme gradient boosting (XGBoost) are commonly used with structured genomic and phenotypic data in crop breeding. RF combines predictions from multiple decision trees and can also provide estimates of variable importance. In tomato (*Solanum lycopersicum*), RF achieved the highest prediction accuracy for fruit weight, fruit width, and pericarp thickness among six models (Yeon et al., 2024). SVM identifies the decision boundary that best separates samples in a high-dimensional feature space, making it suitable for classification and regression with genomic marker data. In alfalfa (*Medicago sativa*), a linear SVM combined with GWAS-selected markers achieved the highest prediction accuracy for fall dormancy and outperformed models based on the full marker set (Zhang et al., 2023). XGBoost builds decision trees sequentially, with each tree correcting errors from the preceding model. In soybean, it performed well across several morphological and seed-quality traits and identified informative genomic regions that partly overlapped with GWAS loci (Gill et al., 2022).

As breeding datasets become increasingly complex, DL has gained attention for its ability to learn informative features directly from high-dimensional data. DL is a subset of ML that uses multilayer neural networks to learn hierarchical representations from high-dimensional data. Compared with conventional ML, it requires less manual feature engineering and is particularly well suited to complex image, sequence, and multi-omics datasets. In digital breeding, DL has been used for high-throughput phenotyping, genomic prediction, trait classification, and multimodal data integration, although its performance often depends on large, high-quality datasets and substantial computational resources. Convolutional neural networks (CNNs), recurrent neural networks (RNNs), and transformers are among the most widely used DL architectures in crop research and breeding. CNNs are well suited to automated phenotyping because they can capture spatial patterns directly from plant images. In lettuce (*Lactuca sativa*), a CNN trained on more than 100,000 labeled signals was used to detect and quantify lettuce heads in large-scale aerial normalized difference vegetation index images, allowing them to be categorized with over 98% accuracy (Bauer et al., 2019). Moreover, deep CNNs were used to quantify pest damage across 231 grapevine (*Vitis* spp.) accessions. These phenotypic measurements were then incorporated into GWAS and GS analyses to identify resistance-associated loci and predict pest resistance (Gan et al., 2025). RNNs are designed to model dependencies in sequential data, whereas long short-term memory (LSTM) networks are better suited to capturing longer-range relationships. In a comparative analysis of 15 genomic prediction methods across six crop datasets, LSTM achieved the highest average performance and ranked first for rice, tomato, and wheat (*Triticum aestivum*) (Pan et al., 2025). WheatGP provides a recent hybrid example, combining CNN and LSTM modules to capture local single-nucleotide polymorphism (SNP) patterns and long-range genomic dependencies, respectively, and achieving high prediction accuracy for wheat yield and several agronomic traits (Wang et al., 2025a). Transformer models use attention mechanisms to capture long-range relationships across an entire input sequence without recurrent processing. GPformer adapted this architecture for genomic prediction through embedding, encoder, and regression blocks, allowing information from SNPs to be integrated regardless of their physical distance (Wu et al., 2024). Across the five crop datasets evaluated, GPformer outperformed the reported baseline models overall. The model was further extended with a knowledge-guided module that incorporated GWAS results as prior information and improved prediction performance in the maize, rice, and soybean datasets (Wu et al., 2024).

### 4.3 XAI models

XAI has gained increasing attention in crop breeding and related research because it helps clarify how AI models arrive at their predictions. Representative XAI studies and their major applications are summarized in **Table 2** and discussed below.

**Table 2:** Applications of explainable and generative AI in crop breeding research.

AI category	Model	Crop	Reference
Explainable AI	SHAP	Almond	Novielli et al., 2024
	SHAP	Apple	Jung et al., 2025
	Cropformer, SHAP	Maize, rice, wheat, foxtail millet, and tomato	Wang et al., 2025b
	IG, LIME, SHAP	Winter wheat	Joshi et al., 2025
	EXGEP, SHAP	Maize	Yu et al., 2025
	Permutation importance, SHAP	Barley	Strudwick et al., 2025
	Grad-CAM, xPlNet	Soybean	Ghosal et al., 2018
	GBP, Grad-CAM	14 crop species	Toda and Okura, 2019
	GBP, Grad-CAM, LRP	Persimmon	Akagi et al., 2020
	Grad-CAM	Rice	Wang et al., 2022
	Grad-CAM, PDP	Citrus	Minamikawa et al., 2022
	Guided Grad-CAM, LRP	Tomato	Akagi et al., 2022
	Saliency map	Soybean	Nagasubramanian et al., 2019
	Attention visualization	Maize, rice, soybean, rapeseed, and brassica	Danilevicz et al., 2023
	DeepLIFT, TF-MoDISco	Tomato, sorghum, and maize	Peleke et al., 2024
	EasiGP	Maize	Tomura et al., 2025
Generative AI	GAN	14 crop species	Bi and Hu, 2020
	SRGAN	Chinese cabbage	Zhang et al., 2022a
	CropPainter	Rice, maize, and cotton	Duan et al., 2022
	CycleGAN	Rice and wheat	Gao et al., 2023
	DCGAN	Rice	Zheng et al., 2024
	CWGAN-GP	Cauliflower, faba bean, and spring wheat	Drees et al., 2024
	ESGAN	Miscanthus	Varela et al., 2025
	FastGAN, Pix2Pix	Barley and maize	Ullah et al., 2025
	FPL-VAE	Grapevine	Miranda et al., 2022
	RVAE, Pix2Pix	Grapevine and cucumber	Benfenati et al., 2022
	GPT-3.5, GPT-4	Wheat and barley	Poretsky et al., 2025
	PlantGFM	12 plant species	Li et al., 2026a

XAI is particularly important in digital breeding, where model outputs need to be linked to biologically meaningful features before they can be used with confidence. Shapley additive explanations (SHAP) and gradient-weighted class activation mapping (Grad-CAM) are two representative approaches. SHAP quantifies the contribution of individual input variables to a prediction and is therefore useful for structured datasets containing genomic, phenotypic, omics, or environmental information. In almond (*Prunus dulcis*), SHAP analysis of an RF model highlighted model-attributed SNPs for shelling fraction, including a highly ranked marker located within a candidate gene that may be involved in seed development (Novielli et al., 2024). Because SHAP attributions can be non-unique among correlated SNPs or markers in linkage disequilibrium, their stability should be assessed and the implicated markers biologically validated. Grad-CAM, in contrast, is mainly used with CNNs to show which parts of an image contribute most strongly to a prediction. In rice, it was used to locate and quantify chalky regions in grain images, supporting high-throughput evaluation of grain quality under high night-temperature conditions (Wang et al., 2022). These methods address different types of breeding data, with SHAP suited to structured variables and Grad-CAM to image-based phenotyping.

### 4.4 Generative AI and foundation models

Generative AI and related foundation-model approaches, which learn patterns from existing data to generate new content or derive useful representations, are increasingly being explored in crop research and digital breeding. Representative studies and their major applications are also summarized in **Table 2**, with further discussion provided in this section. These approaches include image-generative models, text-based large language models (LLMs), genomic foundation models, and emerging agentic systems. These model classes should be distinguished because their training objectives, validation criteria, and potential failure modes differ substantially.

Image-generative models, including generative adversarial networks (GANs) and variational autoencoders (VAEs), have mainly been used to generate, reconstruct, or transform plant images and to augment datasets for downstream phenotyping tasks. GANs are trained through competition between a generator that creates synthetic samples and a discriminator that distinguishes them from real data. For instance, an efficiently supervised GAN (ESGAN), a semi-supervised GAN framework that learns from a small number of labeled images together with large numbers of unlabeled and synthetically generated images, was developed to classify the heading status of field-grown Miscanthus plants from UAV imagery (Varela et al., 2025). The model reduced the requirement for human-annotated training data by one to two orders of magnitude compared with conventional fully supervised approaches. GANs have also been used in multi-stage frameworks. Ullah et al. first used FastGAN, a GAN architecture designed to generate high-quality images from limited training data, to produce synthetic RGB images of greenhouse-grown plants and subsequently applied Pix2Pix, a conditional GAN-based image-to-image translation model, to generate the corresponding binary segmentation masks (Ullah et al., 2025). Across plant species and experimental settings, the predicted masks achieved Dice coefficients of approximately 0.88–0.95 relative to manually annotated reference masks. VAEs map complex inputs into a probabilistic latent space, allowing learned patterns to be reconstructed and new samples to be generated. Miranda et al. applied a fully convolutional VAE with feature perceptual loss to field images of the grapevine cultivars Riesling, Felicia, and Regent (Miranda et al., 2022). The VAE outperformed the autoencoder models used for comparison and achieved an anomaly-detection accuracy of 0.923. Benfenati et al. used a different approach, developing a two-stage framework in which a residual VAE generated new leaf skeletons representing leaf shape and venation, and Pix2Pix converted them into realistic color images (Benfenati et al., 2022). The framework was subsequently used to produce healthy and powdery mildew-affected cucumber leaf images for training a disease-segmentation model. These image-based approaches can be evaluated using reconstruction or generation quality and performance in downstream classification or segmentation tasks. Their limitations include visual artifacts, inadequate representation of biological variation, and the reproduction of biases present in the training data.

Text-based LLMs constitute a separate application class because they are trained on linguistic data and used to extract, summarize, or organize information from scientific literature. In a study of 36 wheat and barley genetic mapping papers, GPT-4 correctly retrieved an average of 80% of the reported traits and 61% of marker–trait associations (Poretsky et al., 2025). Their outputs should therefore be evaluated for extraction accuracy, completeness, consistency, and traceability to the source literature. Potential failure modes include hallucinated information, omission of relevant evidence, and incorrect associations between traits and genetic markers.

Genomic foundation models differ from text-based LLMs because they are trained directly on nucleotide sequences. PlantGFM, a Hyena-based genomic foundation model pretrained on 10.84 billion nucleotides from 12 plant species, supports long-context genomic analysis of sequences up to 64 kb (Li et al., 2026a). Following fine-tuning on annotated plant genomes, it achieved competitive performance in gene structure prediction and enabled the *de novo* generation of candidate plant gene sequences. Although seven selected generated sequences showed transcriptional activity and two showed stable protein expression, these results do not establish their biological function or breeding value. Generated gene sequences therefore require stringent *in silico* assessment and further experimental functional validation. Potential failure modes include biologically implausible sequences, disrupted coding or regulatory features, and poor generalization across species or genomic contexts.

Agentic systems represent a further category in which an LLM interacts with external data, tools, or computational procedures to complete multi-step tasks. Poretsky et al. also evaluated a GPT-based DataFrame agent for filtering and summarizing curated wheat genetic data, providing an early example of this approach (Poretsky et al., 2025). Such systems require validation of the complete workflow, including the accuracy, reproducibility, and traceability of intermediate and final outputs. Because errors can propagate across successive steps, their failure modes may include inappropriate tool use, incorrect intermediate decisions, and unsupported final conclusions.

Collectively, these approaches demonstrate the potential of generative and foundation models in crop research for synthetic image generation, phenotype reconstruction and analysis, literature-based information extraction, genomic sequence modeling, and multi-step data analysis. However, they should not be evaluated using a single common standard, and their direct application to parent selection, crossing design, and cultivar development remains limited.

## 5 Practical applications of AI-driven digital breeding

AI-driven digital breeding is beginning to enter commercial and institutional breeding workflows, although the strength of evidence for its practical benefits varies considerably among applications. Its applications now extend across several stages of the breeding process, from the evaluation and advancement of breeding material to high-throughput phenotyping and the prioritization of parents and crosses. In most cases, AI is not used in isolation but is incorporated into broader workflows that bring together genomic, phenotypic, environmental, and historical breeding data. The examples below therefore range from company-reported operational use and prospective platform development to experimentally validated breeding applications, but few provide independent evidence of realized genetic gain or consistently shortened breeding cycles.

Bayer has described a seed-development pipeline that integrates advanced genomics, phenomics, data science, and AI to support the identification and advancement of promising breeding material (Bayer, 2023). The company also places AI within precision breeding, where it is used to help identify genetic changes associated with favorable or unfavorable plant traits. In this setting, AI contributes to a wider decision-making process that connects large-scale biological data with the selection and testing of breeding materials. Because this evidence is based primarily on company-reported descriptions rather than independently evaluated breeding outcomes, it demonstrates operational interest and integration into commercial workflows, but does not establish realized genetic gain or shorter breeding cycles.

High-throughput phenotyping is one of the areas in which AI has already found a clear practical role. KWS has been developing an image-based system for pepper breeding that uses AI to detect individual fruits, including numerous overlapping fruits within a single image, before conventional image-analysis methods are applied to quantify their characteristics (KWS, 2024). Identification tags allow the measurements to be assigned to specific breeding trials, reducing the need for manual scoring and improving the consistency of phenotypic records. At the time of reporting, the model still required further training for some pepper types and had not yet been implemented across all breeding stations. This example provides evidence of operational development and potential gains in phenotyping efficiency, but not yet of improved selection response or genetic gain. At a larger field scale, TerraSentia, an under-canopy ground robot developed by EarthSense, has been used in commercial maize breeding trials and agronomic studies (DeBruin et al., 2025). Between 2019 and 2023, the platform collected imaging and light detection and ranging (LiDAR) data from nearly 200,000 experimental units, from which computer-vision and machine-learning algorithms generated ground-truth-validated estimates of plant height, ear height, stem diameter, and leaf area index. This represents stronger validation at the phenotyping stage because the derived traits were compared with ground-truth measurements, although downstream effects on genetic gain or breeding-cycle duration were not evaluated. These examples illustrate how AI-assisted phenotyping can support both detailed organ-level measurements and repeated trait assessment across large field experiments.

Public research institutions are also applying AI to crop breeding. For instance, in Japan, the National Agriculture and Food Research Organization used a least absolute shrinkage and selection operator (LASSO) model to predict seed protein content in soybean progeny (Sekine et al., 2021). The model simulated F_2_ progeny from all 18,721 possible crosses among 194 recombinant inbred lines, and the selected combinations were taken through two cycles of crossing and selection. Among the resulting validation lines, six of eight selected for higher protein content exceeded both parents, whereas six of eight selected in the opposite direction showed significantly lower levels. Because model-guided selections were advanced through crossing and phenotypic validation, this study provides experimental evidence that AI-assisted cross selection can shift a target trait in the predicted direction. However, it does not by itself quantify long-term genetic gain relative to a conventional breeding program. An ML-based breeding strategy was also applied to sunflower at Pakistan’s National Agricultural Research Centre (Ibrar et al., 2024). Phenotypic, SSR-marker, and protein-profile data from 109 sunflower genotypes were analyzed using three unsupervised ML approaches. Among these, hierarchical clustering was used to define 12 subgroups, from which 12 high-yielding parents were selected and crossed to produce 36 F_1_ hybrids. Field evaluation subsequently revealed substantial heterosis for seed yield and several other agronomic traits. This provides experimental support for ML-assisted parental grouping and hybrid development, although the contribution of the ML procedure relative to conventional parental selection was not independently isolated.

Beyond these validated applications, public and international breeding institutions are also developing AI platforms for prospective parental selection and cross prioritization. In 2025, the International Rice Research Institute launched the Global AI-Hybrid Rice Platform, developed by its Hybrid Rice Unit in collaboration with the Hybrid Rice Development Consortium. The platform uses AI models to analyze decades of research and trial data together with parental SNP profiles and historical hybrid datasets, with the aim of predicting and ranking F_1_ combinations for particular traits, environments, and market segments (IRRI, 2025). By reducing the number of crosses that need to be produced and tested in the field, the platform is intended to help breeders direct resources toward combinations with greater predicted potential. At present, this initiative should be regarded as a prospective decision-support platform rather than as evidence of realized breeding improvement. The predicted rankings will require prospective validation by producing and evaluating the prioritized F_1_ combinations across relevant environments and comparing their performance with that of crosses selected using conventional breeding approaches. Such validation should determine whether the platform improves prediction accuracy, selection response, genetic gain, or breeding efficiency across completed breeding cycles.

## 6 Conclusions and future perspectives

Digital breeding research is increasingly exploring approaches that connect data generation, model development, selection, crossing, and field validation. Current evidence is strongest for applications such as phenotyping, genomic prediction, and the retrospective prioritization of breeding materials, whereas evidence for fully integrated AI-guided breeding workflows remains limited. As genomic, phenotypic, multi-omics, environmental, and historical breeding resources continue to expand, AI is being investigated for a broader range of roles in evaluating breeding populations, exploring potential crosses, and identifying promising materials at earlier stages. However, many reported applications have been evaluated using historical datasets, simulations, or individual decision points rather than through complete breeding cycles. The long-term value of AI will therefore depend less on the sophistication of individual algorithms than on whether diverse biological information can be converted into reliable and prospectively validated breeding decisions.

A more balanced data foundation will be essential. Genomic resources are generally more extensive and easier to standardize and reuse than crop-specific omics, environmental, and longitudinal field datasets, which remain costly to generate and unevenly represented across crops, developmental stages, locations, and experimental designs. Addressing this imbalance will require sustained investment in field phenotyping, multi-omics profiling, and environmental monitoring. Equally important will be the adoption of common metadata standards, interoperable data formats, and rigorous quality-control procedures that enable records to be integrated across years, sites, and breeding programs. Data provenance should also be preserved so that the origin, processing history, experimental context, and quality of each record can be traced. Without adequate provenance, models may combine incompatible records or reproduce errors and biases that are difficult to identify.

With stronger data resources, predictive models may contribute more directly to breeding strategy by identifying complementary parents, prioritizing prospective crosses, and estimating the performance of untested materials in target environments before extensive field evaluation. Combining genomic information with phenotypic, omics, and environmental data may be particularly useful for complex traits and genotype-by-environment interactions, provided that each additional data layer offers clear predictive or biological value. However, these potential benefits should be treated as hypotheses until model-guided decisions are tested prospectively against conventional breeding approaches and followed through completed breeding cycles.

Generative and agentic AI could potentially extend these capabilities, but their use in breeding remains largely exploratory. Generative models may support data augmentation, the exploration of untested genotype–environment combinations, and the development of alternative crossing strategies. However, they may also generate biologically implausible outputs or reproduce synthetic biases inherited from incomplete or unbalanced training data. Synthetic images, sequences, phenotypes, and simulated populations should therefore be clearly identified, linked to their source data and generating models, and evaluated against relevant genetic, physiological, and environmental constraints before being used in breeding decisions. Generated outputs should not be assumed to represent feasible biological materials merely because they appear statistically realistic.

Agentic systems could coordinate databases, prediction models, analytical tools, and incoming field observations within shared multi-step workflows. At present, however, this remains a prospective rather than demonstrated breeding application. Text-based and agentic systems may hallucinate evidence, select inappropriate tools, misinterpret intermediate results, or propagate errors across sequential tasks. Their use will therefore require transparent audit trails that record data sources, model versions, analytical steps, and consequential outputs. Independent verification and human approval should be retained for decisions involving parent selection, germplasm elimination, crossing, and line advancement. Failure-containment mechanisms should also allow workflows to be halted or reviewed when outputs are unsupported, internally inconsistent, or biologically implausible. Under these conditions, agentic systems may eventually help connect currently separate computational tasks, but their reliability and practical value must first be demonstrated in operational breeding programs.

Translating predictive performance into realized breeding progress is essential to the advancement of digital breeding. Much of the current literature still relies on historical datasets, retrospective validation, or simulation, and relatively few studies have followed AI-selected parents, crosses, or lines through complete breeding cycles. Future studies should prospectively compare AI-guided decisions with conventional selection strategies using predefined evaluation criteria, independent breeding populations, and relevant target environments. They should determine whether AI-guided decisions increase realized genetic gain, shorten breeding cycles, or improve resource efficiency while maintaining performance across environments and preserving genetic diversity. Both successful and unsuccessful predictions should be reported to reduce publication bias and allow the conditions under which models fail to be identified. Returning field observations to the models could support an iterative process of prediction, selection, validation, and refinement, but model updates should be documented and versioned so that changes in performance can be traced to specific data or analytical modifications.

AI is becoming an increasingly important component of selected digital-breeding workflows, but its impact will ultimately depend on the biological and experimental framework in which it is used. Researchers and breeders will remain responsible for defining meaningful objectives, designing representative experiments, generating dependable data, assessing model reliability, and validating predictions under relevant field conditions. They must also determine how improvements in yield, quality, resilience, and adaptation should be balanced against market requirements and the maintenance of genetic diversity. AI-generated predictions and recommendations should therefore support rather than replace biological interpretation and breeder judgment. By combining computational prediction and optimization with biological knowledge and experimental validation, digital breeding may accelerate genetic improvement and cultivar development. Whether these benefits are realized, however, will need to be demonstrated through transparent, prospective, and independently validated breeding outcomes. Sustained collaboration among breeders, biologists, data scientists, and AI researchers will be required to ensure that advances in AI are translated into biologically meaningful, traceable, reliable, and practically effective breeding applications.
